# RosettaRemodel: A Generalized Framework for Flexible Backbone Protein Design

**DOI:** 10.1371/journal.pone.0024109

**Published:** 2011-08-31

**Authors:** Po-Ssu Huang, Yih-En Andrew Ban, Florian Richter, Ingemar Andre, Robert Vernon, William R. Schief, David Baker

**Affiliations:** 1 Department of Biochemistry, University of Washington, Seattle, Washington, United States of America; 2 Interdisciplinary Program in Biomolecular Structure and Design, University of Washington, Seattle, Washington, United States of America; 3 Department of Biochemistry and Structural Biology, Lund University, Lund, Sweden; 4 Program in Molecular Structure and Function, Hospital for Sick Children, Toronto, Ontario, Canada; 5 Howard Hughes Medical Institute, University of Washington, Seattle, Washington, United States of America; University of South Florida College of Medicine, United States of America

## Abstract

We describe RosettaRemodel, a generalized framework for flexible protein design that provides a versatile and convenient interface to the Rosetta modeling suite. RosettaRemodel employs a unified interface, called a blueprint, which allows detailed control over many aspects of flexible backbone protein design calculations. RosettaRemodel allows the construction and elaboration of customized protocols for a wide range of design problems ranging from loop insertion and deletion, disulfide engineering, domain assembly, loop remodeling, motif grafting, symmetrical units, to *de novo* structure modeling.

## Introduction

Computational protein design tools to date have been useful for engineering proteins with a wide range of functions, including DNA binding [1], [2], [3], [4], co-factor binding [5], catalysis [6], [7], [8], [9], fluorescence spectral change [10], peptide-protein specificity [11], [12], and protein-protein interaction [13], [14], [15], [16], [17]. In building nanostructures, computational protein design methods have been applied to designing hyperthermophilic proteins [18], [19], metalloproteins [20], water-soluble membrane channels [21], and higher order macromolecular assemblies [22], [23]. Many of these successes rely on fixed backbone approaches that maintain the backbone conformations seen in the original high-resolution crystal structures and focus on remodeling only the sidechains [18], [24]. In some cases, for example in building coiled-coil structures, an ordered template is often used for designs that contain various degrees of backbone movement [25].

Flexible backbone protein design requires energy functions of sufficient accuracy and sampling methods of sufficient power to allow prediction of the backbone structure that a remodeled section of the protein chain is likely to adopt. The Rosetta energy function and sampling methodology, although far from perfect, have shown considerable promise for protein structure prediction and hence are reasonably well suited to flexible backbone protein design [8], [26]. This is illustrated by the successful design of a 10 residue protein loop [27], a 16 residue helix-loop segment contributing to a protein core [28], a protein-binding peptide [29] and a very stable protein with a novel protein fold [30], all of which achieved atomic level accuracy.

**Figure 1:**
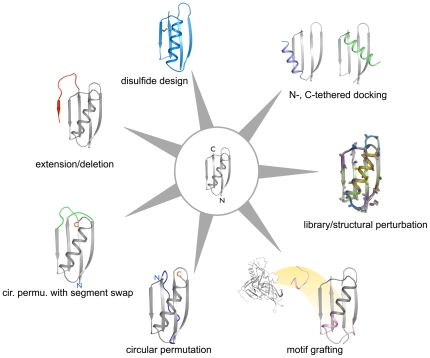
Examples of backbone manipulation using RosettaRemodel. In the center is the crystal structure of protein G (PDB ID: 1PGA), which was used as the starting point for all the different cases. The colored regions highlights changed made with RosettaRemodel.

We describe here a versatile protocol, RosettaRemodel, that combines the tools in Rosetta to address a wide range of problems in flexible backbone design. RosettaRemodel utilizes the (1) native protein parameterized Rosetta force field [31], (2) fragment-based structural building from Protein Data Bank (PDB) torsion angles [26], [32], (3) robotics-inspired chain closure algorithms [33], [34], (4) iterative approaches for searching the sequence landscape [27], and (5) short folding simulations for design validation. Figure 1 shows some examples of flexible backbone designs that can be carried out using RosettaRemodel.

## Results

### Applications to Date

RosettaRemodel has been applied to a number of design problems with positive results: a beta-knee was designed on integrin with various lengths to understand its activation [35]; a protein antigen of known structure was circularly permuted with a loop insertion linking the N- and C-termini, and a crystal structure solved for the circular permutant agreed well with the best model over the designed loop [36]; sequences from selection experiments on a DNA binding protein were modeled to deduce terms that would correlate computational models with experimental selections; a model of human granulocyte-macrophage colony-stimulating factor (hGM-CSF) fusion to HIV GP120 spike was built to illustrate structural compatibility [37]. Many other applications of RosettaRemodel are still being tested; the remainder of this article illustrates the diversity of problems that can be addressed.

## Design and Implementation

### Blueprint Interface

**Figure 2:**
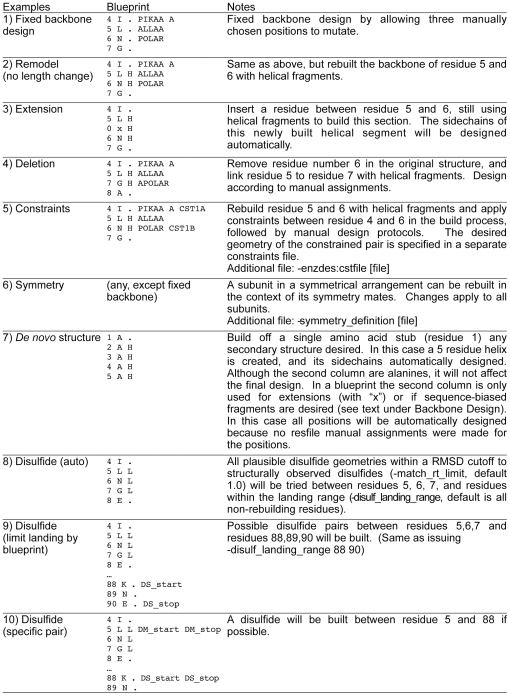
Blueprint Assignment Examples.

The RosettaRemodel protocol uses a simple interface, the *blueprint*, and a number of run-time switches to mediate various protein modeling tasks. Examples from blueprint files for different tasks are shown in Figure 2; each example will be addressed in the text below. A blueprint file handles backbone building, sidechain design, disulfide pairing, and at-build-time constraint assignments if needed. Its layout allows one to easily understand all the operations done to a structure. In a column layout, each line in the blueprint represents a residue in a structure. For backbone remodeling, residues can change their secondary structure, be deleted, or be created according to blueprint assignments. For sidechain perturbations, the blueprint allows all the operations in RosettaDesign. With these features, RosettaRemodel in most cases will only need a blueprint and a starting PDB file to carry out design tasks that involve the backbone, sidechains, or both.

### Setting up RosettaRemodel

RosettaRemodel takes an input PDB file (-*in:file:s*) and a blueprint file (-*remodel:blueprint*) to carry out most of its functions. Although not required, it is recommended that the residues in the PDB file be renumbered starting from one before creating a corresponding blueprint file. Due to the layout in columns in the blueprint file, using editors that allow text column manipulation, such as *vi* [38], makes setting up remodeling a relatively simple task.

### Sequence Design

When only sidechain design commands are given in the blueprint file, RosettaRemodel handles fixed-backbone design in a similar manner as RosettaDesign. Sidechain mutations or rotamer re-sampling can be achieved using all the commands available to a RosettaDesign resfile, such as PIKAA, ALLAA, APOLAR, EX1, EX2, etc. For an example see Figure 2, section 1. However, when accompanied by RosettaRemodel specific flags: *-find_neighbors* and *-design_neighbors*, neighboring residues will be automatically selected following RosettaRemodel specifications (6 Å). With -*find_neighbors* alone, neighboring residues will be repacked without altering their amino acid identity; in combination with -*design_neighbors*, all these positions will be designed for the most suitable amino acid. Due to the stochastic Monte Carlo process used in sidechain selections, normally one would create multiple design runs to ensure convergence in the search process. With RosettaRemodel, this step can be customized by setting the number of trajectories to try (-*num_trajectory*) and the number of lowest energy models to output (-*save_top*), so that a reasonable amount of Monte Carlo sampling can be carried out but only the few decoys with lowest energies will be output. This principle is used for all RosettaRemodel builds – flexible or fixed backbone – as RosettaRemodel internally screens structures during a trajectory by their energies.

In addition to the manual assignments described above, residues are processed automatically according to the number of surrounding residues and use only a subset of amino acids that fits the description. Since computational methods are most reliable in designing the core of a protein with rotamer packing, RosettaRemodel uses core residues alone to bias the simulation for a better hydrophobic core. This is achieved by ranking the degree of exposure for each of the residues involved and reducing contributions from the highly exposed ones. By masking these positions – temporarily switching them to alanines – the design trajectory can be directed to favor interactions in the core. The automated protocol postpones the decision on designing the fully exposed positions until reasonable hydrophobic support has been built, before the final output. The protocol accumulates low energy designs solely based on hydrophobic packing in a trajectory before designing surface residues. Although exposed polar residues are important for stability, modeling them accurately has been shown to be difficult as RosettaDesign lacks electrostatic energy terms for general applications. This layered treatment, however, is not applicable to all situations, and can be turned off by the flag:*-skip_partial*.

### Backbone Design

New backbones are built up from fragments of specified secondary structure [8], [26], [28]. The secondary structure is specified by the third column of any position in the blueprint file: when given an assignment of H, L, E, or D, which stands for helix, loop, extended strand, and degenerate (random), respectively, the corresponding position will be rebuilt with the specific fragment type chosen. For an example see Figure 2, section 2. If the sequence is known for the segment to be built, as is the case when predicting the conformation of a known loop, one can manually assign the positions with PIKAA commands to their native amino acids. In a prediction case, one can also bias the fragment-picking process by giving preferences to fragments that share identity to some or all of the positions, following the sequence given in the second column of the blueprint file. This can be achieved by using the flag: *-use_blueprint_sequence*. This process, however, does not provide secondary structure prediction based on sequence – the fragment types must be manually assigned and will be strictly followed. Successful prediction using this functionality will usually involve strong sidechain-driven structural features and relies on successful full-atom refinement to model the site.

### Implementation of Backbone Remodeling with Direct Fragment Generation

RosettaRemodel harvests fragments directly from a culled set of torsion angles from non-redundant x-ray structures and assembles them on the scaffold structure according to their secondary structure type. An advantage of harvesting fragments directly is that one can collect a new set of fragments during a protocol and not be limited to the pre-defined set provided at the start of the simulation, resulting in significantly expanded sampling diversity. The default number of fragments used is 200 segments of nine-, three-, and single amino acids for each position, as is commonly used for other Rosetta fold prediction projects [31], [39]. Additionally, the protocol allows harvesting fragments that match the entire length of a remodeled region, potentially with improved fragment qualities similar to those previously reported [40]. Since the objective is to design new structures and new sequences, all force field terms that involve specific sequence information are explicitly turned off. Only van der Waals, radius of gyration, and Ramachandran probability terms are used to specifically address clashing, packing, and chain geometry, respectively. Residues in the backbone building stage are centroids of valines or alanines, and thus the Ramachandran term is the same for all moving positions, but is significantly scaled down to 1/10 its normal weight to avoid areas of very low probability.

Backbone modeling on internal loops is performed with random cut sites within the loops preceding fragment building. The internal chain breaks are subsequently reconnected using closure algorithms such as Cyclic Coordinate Decent (CCD) [33] or Kinematic Closure (KIC) [34]. Only models with properly closed chains after the fragment assembly stages are passed along to the design stage. There are often cases where successful closure is rare; in such cases it may be that too few residues are being used or that the residues at the ends of the loop being modeled are in orientations not suitable for proper closure and should be allowed to move.

Forcefield terms that enforce backbone geometry can be applied and adjusted to suit particular design problems. Backbone-specific terms, namely strand pairing and hydrogen bonding energies on helices and sheets, can be selectively applied for different types of designs; conversely, the terms used by default can be scaled down or turned off for purposes such as turning off minimization of the radius of gyration when building a polar surface loop. We use either centroids of valines or alanines in the fragment assembly stage as generic space fillers until the design takes place at the full-atom level. Although contacts between sidechains are evaluated and are part of the Monte Carlo simulation when sampling backbone conformations, evaluation of proper chain closure supersedes all other criteria.

### Implementation of Trajectory Accumulator for Multiple Objective Optimization, Clustering, or Checkpointing

RosettaRemodel tracks structures it has built internally. This structure accumulation stage has three different purposes. First, it allows one to use a primary score to collect sorted structures from the full-atom design step following the centroid building step, and subsequently filter or find unions with other criteria as a simple multiple objective optimization tool. Second, the structures collected can be clustered into groups of unique conformations if *-use_clusters* is set true. The search strategy in this case is to first perform massive random sampling of different regions of the folding landscape by large fragment-based moves, and once the cluster centers are identified, then focus on refining the unique structures by localized sampling. Third, the sorted list of structures will always contain the best answer in the trajectory at a given time. A convenient checkpoint scheme (-*remodel*:*checkpoint*) is built into this protocol by maintaining the candidate list on disk.

### Iterative Design and Refinement Optimization

Models listed in the accumulator from a centroid building stage can be subjected to a number of iterative design and refinement cycles. For speed or other considerations, this can be bypassed by issuing -*remodel:quick_and_dirty* flag, which will make models only from fragment insertion without fine-tuning the backbone geometry for new sequences. Refinement steps rebuild backbones with either CCD (default) or KIC, and these backbone altering steps are followed by sidechain designs according to user's choices as described previously in Sequence Design section. This cycle iterates three times by default (or more, as specified by *-dr_cycles* flag with an integer). This iterative design-refinement step, in conjunction with the trajectory accumulator, allows a more focused exploration of the sequence landscape by applying time-consuming refinement only to structures ranking well from centroid stages or cluster centers that are unique in structures.

### Extensions and Deletions

Simply adding and subtracting lines from blueprint files will create a new structure that follows the corresponding actions. For examples of extensions and deletions in the blueprint file, see Figure 2, sections 3 and 4. When inserting a residue, a line in the blueprint starting with “0 x” will cause insertion of one residue at the corresponding position in the structure. By assigning secondary structures to the segments and the regions flanking the modified region, one can conveniently alter the length of a protein chain. These are implemented using fragment building as described above

### Constraints

**Figure 3:**
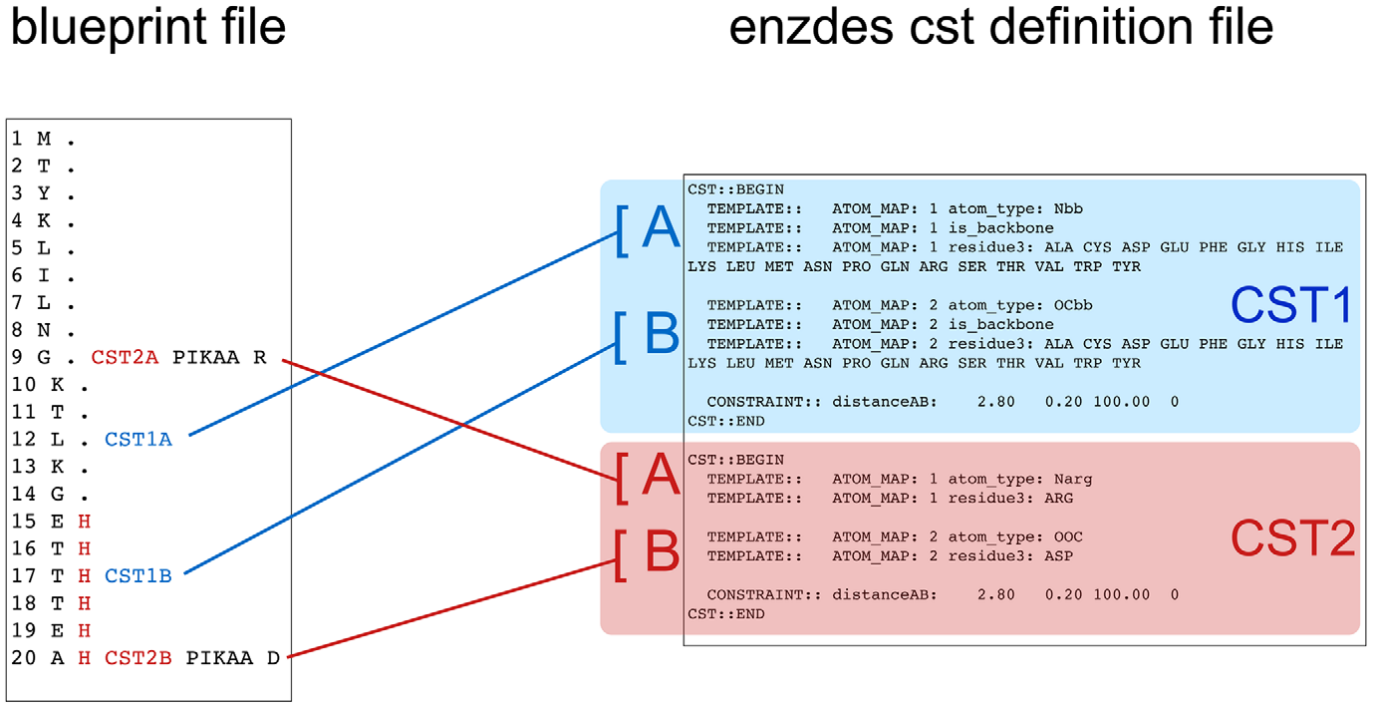
Constraints file and its associated blueprint file. Each block in the constraints file is represented in the blueprint file with the CST1A, CST1B, CST2A and CST2B notation. The enzdes constraint format is discussed in Richter et al. [41] in detail. In this figure we show the association between cst and blueprint files. Each enzdes constraint block, defined between CST::BEGIN and CST::END statements, always contains two elements, and to interface with blueprint, the first definition is defined as “A” and the second as “B,” and each element requires a corresponding assignment in the blueprint. Each block is also associated with a numerical value from 1 to the total number of blocks defined in the cst file. In this example, the first constraint pair (CST1A/CST1B) is used to restraint one of the residues (residue 17) on the strand being built (residues 16–20) to within a hydrogen bonding distance with a stationary residue (residue 12). The second constraint pair (CST2A/CST2B) operates on the sidechains of residue 9 and 20 for hydrogen bonding between the functional groups. The distinction between a backbone and sidechain definition is the choice of atom types using Rosetta atom type names and a required “is_backbone” statement because enzdes constraint protocol does not automatically treat atoms as backbone by names. In this example, the hydrogen bonding constraint is defined for a pair of atoms within 2.8+/−0.2 Å, with a force constant of 100. The trailing “0” in the distanceAB definition is for non-covalent interaction.

**Figure 4:**
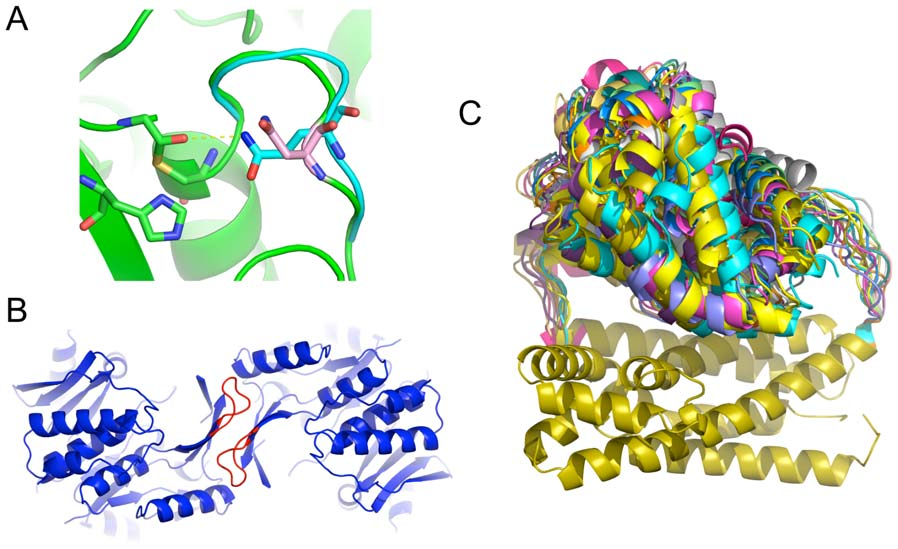
Examples of designs made with RosettaRemodel. A) A cysteine protease site, with cys-his intermediate shown in green sticks. The loop in cyan was rebuilt from its original position (in green) to introduce sidechain-directed stabilization of the oxyanion in the intermediate. The re-designed model uses an asparagine in direct contact with the oxyanion instead of the wild-type aspartic acid (in pink). B) Two interacting loops in a symmetry arrangement were rebuilt to increase interactions across the subunits. C) Domain localization. A domain assembly designed with a pair of linkers. In this figure, the ensemble of an internally inserted domain is shown moving relative to the stationary structure that hosts the insertion as a result of sampling the loops linking them. With this type of sampling, one could model the localization of the final assembly.

RosettaRemodel handles constraint assignments together with length changes in the blueprint file. A separate constraint definitions file used to describe the atoms involved and the geometry required can be generated without specifying the residue positions, therefore allowing the same set of constraint definitions to be used for different designs with varying chain length. RosettaRemodel uses the constraint file format from the Rosetta enzyme design protocol [41], allowing constraints to be specified in up to six degrees of freedom. An example of a constraint file and its corresponding blueprint are given in Figure 3 (a constrained blueprint is also shown in Figure 2, section 5), where two constraint blocks were defined to form a hydrogen bonding pair with distance constraints. In Figure 4A, we showed an example of modeling a sidechain to satisfy an oxyanion intermediate on a cysteine protease active site with RosettaRemodel and constraints.

To use constraints in remodeling, one can specify if sidechain or backbone atoms are involved and those constraints will be applied in different building stages. During fragment insertion, all sidechains are represented by default as centroids of valines (but can be changed to alanine centroids), so only backbone constraints will be used. Constraints involving sidechain atoms will only be used during rotamer optimization. To satisfy a constraint involving at least one atom on a sidechain, it is advised that the constraint setup have an accompanying definition for their backbones. For example, if a hydrogen bond donor on a sidechain is to be constrained to an acceptor, in addition to the description of the sidechain hydrogen bond, a rough distance constraint can be assigned to the backbone atoms of the donor residue so that during the backbone building stage the residue would be brought to the proximity of the acceptor. This would maximize the chance of building the donor sidechain with the desired geometry.

### Symmetry

By giving RosettaRemodel a symmetry definition file [42], one can simultaneously remodel all the subunits in a symmetrical assembly. This setup allows users to take advantage of all of RosettaRemodel's functionality to build sections of a subunit that interact with other subunits in a symmetrical arrangement. The blueprint used for these cases only needs to describe a single subunit; symmetry related units will be automatically generated and output. In Figure 4B, we show building a loop-mediated interaction across a dimer interface. In this model, strands on the starting template were extended to allow new contacts to be introduced across the interface. The loops in red were built simultaneously obeying symmetry.

### *De novo* Structure Modeling

Because RosettaRemodel selects fragments only by their secondary structures following the user's suggestions, it is straightforward to build *de novo* structures. In Figure 2, section 7, by asking for a string of helical fragments, one can build an ideal helix that could be used for other remodeling purposes. If building a specific topology out of secondary structure elements and the lengths of the building blocks are known, one can build a protein *de novo*, much like that of Top7, a previously reported protein with novel topology [30].

### Disulfides

Although improving packing has been shown to achieve improved stability, in some cases a disulfide linkage is a better alternative. RosettaRemodel offers two different ways of engineering disulfides: (1) building disulfides in the native environment with minimum perturbation, if a realistic disulfide can readily be made with the native backbone, and (2) aggressively rebuilding the backbone until disulfide geometries can be satisfied. The difference is the inclusion of fragment insertion steps, controlled by a flag: -*bypass_fragments*. Disulfides are treated as connecting a mobile region to a stationary region. To build disulfides from one region of the structure to the other, a range of mobile positions should be designated for rebuild in a blueprint file by assigning the desired secondary structure types, and the flag -*build_disulf* must be issued. The entire rebuilding (mobile) range will be considered for disulfide building; this range can be further restricted by tagging a subset of the movable positions with “DM_start” and “DM_stop” tags in the blueprint file – these tags stand for “disulfide mobile start” and “disulfide mobile stop”, respectively. The stationary positions considered for disulfide design default to all positions not designated for backbone movement and can also be narrowed down to more specific regions by either tagging two positions in the blueprint as “DS_start” and “DS_end”, or using the flag -*disulf_landing_range* followed by two integer numbers. Giving the same position as both the start and end position will restrict samping of disulfides only to this position. See Figure 2, sections 8, 9, and 10 for examples. The engineered disulfide will always connect one position in the specified rebuilding (mobile) region with one position in the specified stationary region. All position pairs in the specified regions with their Cβ atoms within 5 Å of each other will be checked for disulfide geometry. If multiple pairs satisfy the Cβ distance check in one structure, all are considered and handled by the structure accumulator described previously. To assess candidate disulfide geometries at a higher resolution than Cβ distance, RosettaRemodel compares a pair of protein backbones with realistic disulfide geometries described in a database and returns a RMSD score. A threshold value can be set (*-match_rt_limit*) to allow distant matches to be tested; if this threshold is set too low, RosettaRemodel may not find any candidate position pairs. Once a pair of positions has been identified, the residues at those positions will be mutated to cysteine on the all-atom level, and series of subsequent minimization steps will optimize the disulfide geometry before continuing the rest of the RosettaRemodel protocol.

### Advanced Functionality

#### Domain insertion and motif grafting

A structure segment can be inserted unmodified to another protein, transplanting a linear structural motif, an ideal structure, a domain, or an entire protein. The file with the structure to be inserted in PDB format should be edited to contain only the section of interest. Contents in the file will be processed by RosettaRemodel with the invocation of the flag -*insert_segment_from_pdb*, followed by the filename. The insertion should be described in a blueprint file as part of a rebuilding segment, but rather than being assigned for rebuild with secondary structure notations H, E, L or D, the inserted segment is designated with “I”, for insertion. The number of residues with insertion designation should match the number of residues in the inserting file. The range of the insertion should be flanked by several rebuilding residues to maintain proper connection with the rest of the structure. The number of residues to be used as the linker is usually determined by trial and error.

#### Tethered docking

In certain cases, the polypeptide chain can be gradually built out from one end of the structure to the other, often guided by loose constraints instead of chain closure. The stringent requirement for chain closure by RosettaRemodel may sometimes compromise secondary structure integrity during a simulation if an erroneous chain length is used. Predicting *a priori* the optimal chain length is difficult and is best obtained by trials with large sampling. This, however, requires much computing time. Instead, if the objective is to optimize packing of a helical segment with its neighbors, one can first optimally dock the helix in a tethered fashion to the site to satisfy features described by the environment. Once the helix is placed in the structure, the chain can grow out further in the next iteration until it is fully connected with the rest of the structure. To sample such processes, each intermediate step can use the flag *-bypass_closure* to turn off the chain closure requirement for that step.

#### Domain localization test, domain assembly, tethered docking and circular permutation

RosettaRemodel can be used to sample the degrees of freedom when engineering protein fusions with linkers, and this can be extended to sample domain assembly problems where a known linker is between two rigid body domains. For a simple linear two-domain assembly [43], one can simply define the linear region with its appropriate secondary structures and run RosettaRemodel with the *-no_jumps* flag to fold through the linker to sample the degrees of freedom. We offer here a recipe for a more complicated scenario that combines the use of several features in RosettaRemodel. In collaboration with Goreshnik and Maly [44], we used RosettaRemodel to sample linker lengths to optimally position inhibition domains, shown in Figure 4C. In order to create the linear fusion of BclxL-DH-BH3 while maintaining the dimer structure of BclxL and BH3, the PDB of BclxL/BH3 complex was first renumbered as a single chain, ignoring the chain break that was originally the termini for the two molecules, making a new PDB with the N-terminal leading into BH3 and C-terminal exiting out of BclxL. The DH domain was then similarly circularly permuted with a text manipulation script to move its original N- and C-termini internal to the sequence. With these permutations, the BclxL/BH3 complex could be inserted in between the original N- and C-termini of DH using the domain assembly scheme describe above and effectively created the BclxL-DH-BH3 fusion. A number of linker lengths were sampled in order to position the BclxL/BH3 complex atop the binding site of DH. This could be done directly using chain closure to model all three molecules together such that only linkers sufficiently long will yield results, or it could be done in the step-wise fashion as discussed in the tethered docking section so only one linker was built at a time. Superposition of the models generated provided information on the degree of freedom of this assembly, and indeed for our test built with the linker sizes chosen, we modeled the BclxL/BH3 complex atop of the DH binding site for steric occlusion/inhibition.

#### Local sampling and focused library generation

A model or a starting structure can be subjected to a number of design-relaxation refinement cycles to sample local conformational spaces and variations in sequences. This could be done to optimize the sequences for a design or for the purpose of collecting sequences compatible with the topology to make focused experimental libraries. For local structural changes, fragment insertion can be skipped by using the flag -*bypass_fragments*, and instead of running the default CCD based refinement, the relaxation step is run using the Rosetta Relax protocols by issuing -*run_pose_relax* flag. When these two flags are used, the secondary structure designation in the blueprint file is no longer relevant; the H, L, E, or D assignments become equivalent and only provide information on whether a residue is restrained. In RosettaRemodel the structural relaxation stage is further restricted by automatically restraining positions untouched in the blueprint file to their starting position. One can also specify the number of design-relaxation cycles to be used (default is three) by issuing the -*dr_cycles* flag, followed by an integer number.

#### Structure prediction and validation of remodeled sequences

Structure prediction starting from amino acid sequences alone requires deducing possible secondary structures from the linear sequence, and RosettaRemodel should not be used for predicting structures from primary sequences as it does not handle secondary structure prediction information. In the context of sampling conformations of a short range of residues within a protein, however, RosettaRemodel can take advantage of the conformational sampling steps to achieve reasonable “structure prediction” results by exploring the energy landscape around the residues of interest. Structure prediction in this sense can be considered a subset of design because both require the build stages and only the sequence is invariant for predictions. By assigning each position its final amino acid designation through the PIKAA command in the blueprint, a structure prediction run can be carried out, relying largely on full-atom refinement. If the second column of the blueprint file is left as the native sequence, one can also bias fragment picking to favor fragments that share common amino acid residues with the corresponding sections using the flag: -*use_blueprint_sequence*. The user can use the default CCD refinement protocols or alternatively switch to using KIC with a flag (-*swap_refine_confirm_protocols*) to use the method as described in Mandell, et. al. [34].

We tested this functionality of RosettaRemodel on a set of 40 proteins with eight residue loops. Since the full set of sequence-dependent scoring functions normally used for prediction is not part of the design toolset, we do not expect results to match those of the prediction runs. For benchmarking if native structures can be recovered largely by full-atom refinement alone, we did not bias fragment selection based on sequences – RosettaRemodel used only loop fragments harvested randomly for each trajectory. Nonetheless, we expect reasonable performance and indeed that is what we find. The Cα RMSD distribution of the models produced by RosettaRemodel are below 2 Å deviation for the majority of the cases, only slightly worse than the reported values [39].

Several different versions of the build protocol were tested against the eight-residue loop set, and a correlation was observed between the convergence of the two closure algorithms and the predicted Cα RMSD against native structures. While this correlation should not be considered as the definitive measure in picking out models for experimental testing, it does provide a qualitative measure of the loops generated, and RosettaRemodel reports these values for reference. We noted that the correlation with RMSD to native was only observed when KIC was applied after structural refinement with CCD. We observed little advantage of KIC over CCD in the iterative building-refinement stage. Therefore the algorithm is setup to iteratively design and refine using the CCD algorithm, and only before the final model is generated will an optional KIC refinement stage be applied.

### Conclusion

RosettaRemodel was originally created to handle structural design problems involving flexible backbones, and was further extended to handle a wider variety of design problems. It offers a unified ‘blueprint’ interface for many design scenarios which can conveniently access a range of Rosetta functionalities. Currently there are a number of tools using Rosetta for structure manipulation, ranging from the fully interactive FoldIt [45] to the fully automated RosettaScripts. RosettaRemodel is semi-interactive because it relies on the user to provide a sensible blueprint for the simulations and it often requires a few iterations of user modifications to the blueprint before a good setup is found. Although RosettaRemodel describes a fully self-contained protocol, it is sometimes desirable to use it in the RosettaScripts setup to take advantage of other specialized protocols, and this is indeed possible.

The build examples described here are the general problems that can be addressed using RosettaRemodel. Several cases can be combined into one remodeling step or used in separate steps to build a structure that meets the desired specifications. Flexible backbone design problems are difficult and RosettaRemodel aims to provide a convenient way to address them.

## Availability and Future Directions

RosettaRemodel is part of the Rosetta molecular modeling suite, available through http://www.rosettacommons.org/. A multi-threaded structure accumulator is being developed for RoesttaRemodel to facilitate runtime efficiency and potentially incorporate mechanisms for multi-state designs. A front-end user interface using FoldIt to facilitate annotating and generating blueprint files is also being planned, such that designs can be carried out in a single interactive environment and not depend on text manipulation and external visualization processes.
